# Intellectual capital in the healthcare sector: a systematic review and critique of the literature

**DOI:** 10.1186/s12913-015-1234-0

**Published:** 2015-12-15

**Authors:** Jenna M. Evans, Adalsteinn Brown, G. Ross Baker

**Affiliations:** Institute of Health Policy, Management & Evaluation, University of Toronto, Toronto, Canada

**Keywords:** Intellectual capital, Organizational knowledge, Intangible assets, Knowledge management, Health system performance

## Abstract

**Background:**

Variations in the performance of healthcare organizations may be partly explained by differing “stocks” of intellectual capital (IC), and differing approaches and capacities for leveraging IC. This study synthesizes what is currently known about the conceptualization, management and measurement of IC in healthcare through a review of the literature.

**Methods:**

Peer-reviewed papers on IC in healthcare published between 1990 and 2014 were identified through searches of five databases using the following key terms: intellectual capital/assets, knowledge capital/assets/resources, and intangible assets/resources. Articles deemed relevant for inclusion underwent systematic data extraction to identify overarching themes and were assessed for their methodological quality.

**Results:**

Thirty-seven papers were included in the review. The primary research method used was cross-sectional questionnaires focused on hospital managers’ perceptions of IC, followed by semi-structured interviews and analysis of administrative data. Empirical studies suggest that IC is linked to subjective process and performance indicators in healthcare organizations. Although the literature on IC in healthcare is growing, it is not advanced. In this paper, we identify and examine the conceptual, theoretical and methodological limitations of the literature.

**Conclusions:**

The concept and framework of IC offer a means to study the value of intangible resources in healthcare organizations, how to manage systematically these resources *together*, and their mutually enhancing interactions on performance. We offer several recommendations for future research.

**Electronic supplementary material:**

The online version of this article (doi:10.1186/s12913-015-1234-0) contains supplementary material, which is available to authorized users.

## Background

As with other organizations, among the most valuable assets of healthcare organizations are the knowledge, skills, and experiences of their leaders and professionals. These intangible resources, coupled with the value derived from internal capabilities and external relationships, constitute the *intellectual capital* (IC) of healthcare organizations and systems [1]. Healthcare organizations possess vast structured and unstructured stockpiles of formal and informal know-how distributed across the minds of individuals, captured in files, databases, and reports, and embedded in the culture and routines of organizations themselves. An organization’s IC also extends beyond its boundaries encompassing external elements such as partnerships, stakeholder relations, and brand or reputation in the community.

Variations in the performance of healthcare organizations may be explained, in part, by differing “stocks” of IC, and differing approaches and capacities for leveraging IC [2]. Although healthcare is a knowledge-intensive industry, few healthcare organizations systematically and strategically manage their IC to meet strategic goals and improve performance [3, 4]. Because IC cannot be observed and understood in the same way as financial and material capital, it is rarely systematically measured and monitored in meaningful ways that go beyond the use of rough indicators that tend to focus on training. This gap in measurement creates six inter-related challenges for managers and policy-makers.

First, managers and policy-makers often have difficulty assessing whether necessary human resources, capabilities and processes are in place for the successful development and implementation of strategy, change or innovation. A lack of awareness or understanding of the IC flowing through an organization or system threatens the success and sustainability of new initiatives, and the efficacy of strategic planning, decision-making, and change management.

Second, there is a growing volume of data, information, and evidence on healthcare policy, management and delivery, but it is often not put into practice efficiently and effectively. This explicit knowledge, paired with the constant flux of tacit knowledge within organizations, raises the question of how to filter, extract, integrate and deploy IC at the point of execution.

Third, IC is highly portable and tacit, and therefore often lost through employee attrition and turnover, improper documentation, and major restructuring. Workforce instability among leaders and front-line staff, the limitations of administrative and clinical databases for supporting research and learning, and demands for large-scale reform are common challenges facing healthcare organizations and systems. Furthermore, physicians are often not direct employees of healthcare organizations and may have little interest in contributing their time and knowledge to organizational improvement efforts. Without stronger management of such issues, what is known (or could be known) at one point in time is lost with the passage of time.

Fourth, leaders of healthcare organizations must respond to multiple stakeholders and meet performance goals across multiple – often competing – dimensions of effectiveness, including access, quality and cost. In addition, many healthcare organizations, such as teaching hospitals and regional planning and governance bodies, have missions that include knowledge generation, translation and application in addition to service delivery and system planning respectively. The concept of IC offers a broad lens for identifying and examining the resource configurations that best support achieving the complex mandates of healthcare organizations.

Fifth, there is an increasing divide between how innovation and evidence are measured, managed and supported in healthcare systems. Despite their mutually reinforcing agendas and importance to improving performance, separate structures and processes often exist to support innovation and evidence. Examples include organizations with a Chief Innovation Officer and a Chief Quality Officer who operate largely in isolation of one another, and government agencies that lack an integrated approach to decision-making on innovation, quality, technology and risk. Healthcare systems and organizations require a framework for managing all of these together.

Finally, healthcare delivery is very siloed which makes it difficult to use available IC across an organization and among organizations. These silos include major divisions between clinical and managerial knowledge and between acute care and community care knowledge, despite the fact that they may describe the same conditions and the same groups of patients. The challenges associated with integrating and leveraging diverse stocks of IC from across the healthcare system limit the efficacy and impact of system-level reform efforts that rely on the buy-in and alignment of multiple stakeholder groups. For example, policy or organizational changes to enhance access, quality of care, and integration requires the recognition and use of IC from *across* the healthcare system. Methods for understanding, monitoring and leveraging IC are needed, whether the aim is to transfer best practices across organizations and regions, to coordinate the knowledge and capabilities of diverse providers, or to determine where to make investments for maximum impact.

The management challenges outlined above, paired with the dynamic and highly politicized healthcare environment, create a unique setting within which to apply an IC perspective. However, the explicit application of an IC lens to the healthcare sector is relatively new and has not been rigorously examined conceptually or empirically, nor has the work to date been synthesized and critiqued to determine quality and generalizability. The aim of this paper is to synthesize what is known about the management and measurement of IC in healthcare organizations through a review of the literature. The following research questions guided the study: (1) How is IC conceptualized and defined in healthcare organizations? (2) How is IC measured in healthcare organizations? (3) What is known about the relationship between IC and performance in healthcare organizations, and management efforts to improve performance through IC?

## Methods

In consultation with a library scientist, we devised a list of terms to search several electronic databases for peer-reviewed academic literature. A broad list of search terms was tested to identify terms that produced relevant articles and minimized redundancy. Based on these preliminary searches, and given the use of differing terms to refer to IC, searches of title, abstract and/or keyword fields were conducted using the following search terms: intellectual capital/assets, knowledge capital/assets/resources, and intangible assets/resources. These terms were paired with health care/service*, hospital, public health, medic* and nurs*. IC emerged as a concept in the early 1990s [5]; we thus searched for articles published between 1990 and 2014. Several databases were searched including PubMed, CINAHL, Business Source Premier, Web of Science, and Scopus. We limited the results to papers published in academic journals. The reference lists of included papers were also reviewed to identify additional relevant papers.

Papers had to meet the following criteria for inclusion: (a) written in the English language, (b) published in a peer-reviewed academic journal, (b) focuses on identifying, managing or measuring intangible organizational resources, (c) considers at least two of the three types of IC (human, structural, relational), and (d) focuses on healthcare delivery organizations. To increase the relevance of selected papers to our research questions, we applied several excluding criteria. First, papers explicitly focused on knowledge management, organizational learning, or human resources management – with no discussion or application of the concept of IC – were excluded. Although these concepts are related, they represent distinct bodies of literature with differing foci and existing reviews. Some papers were narrowly focused on human resources issues, but used the term IC and drew from the IC literature; these were included because of the search strategy. Some papers did not use the term IC, but their conceptualization of similar concepts, such as “organizational knowledge” and “intangible assets”, align with the IC definition and framework and were thus included (e.g., [6, 7]). Second, papers on pharmaceuticals, biotechnology, and technology/equipment/supplies companies, or on education and research institutions (but not teaching hospitals or academic medical settings), were excluded. Organizations that deliver healthcare services (e.g., hospitals, primary care practices, long-term care homes) have multiple, conflicting missions, unique management challenges, and different performance measures than those producing products and technologies or those delivering educational services. To enhance comparability and generalizability, only papers focused on healthcare delivery organizations were included. Third, papers focused exclusively on web-based knowledge systems for clinical training and decision-making – with no discussion or application of the concept of IC – were also excluded. Although these systems represent a form of structural IC and influence human IC, these papers tend to focus on system design, user satisfaction, and/or system impact, not on the value-add of the tools and systems to the organization’s IC. Finally, commentaries and editorials were also excluded along with grey literature such as conference papers and reports. As part of the preliminary searches conducted to test search terms and strings, we evaluated a sample of conference abstracts (*n* = 25) and, where available, full conference papers (*n* = 15). Due to inaccessibility and poor quality in terms of content, contribution to knowledge, and writing style, the scope of the review was limited to peer-reviewed published papers. Studies were not excluded based on their methods, but we did assess the methodological rigour of included studies.

Articles deemed relevant for inclusion underwent systematic data extraction, using a data extraction form developed by the research team, to identify overarching themes. We extracted descriptive characteristics including study purpose, study context, definitions of key concepts, theoretical framework, methods/approach, and key findings or main points. We also conducted a bibliometric analysis of included papers to help us characterize the literature. The methods and results of the bibliometric analysis are provided in Additional file 1. The review methods and final report herein were conducted in accordance with the “Preferred Reporting Items for Systematic Reviews and Meta-Analyses” (PRISMA) guidelines. A PRISMA checklist is provided in Additional file 2.

## Results

**Fig. 1:**
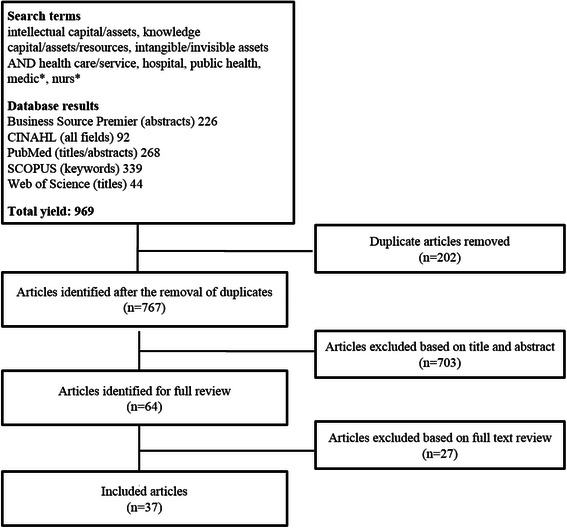
Flow chart of search strategy

The search strategy produced over 750 bibliographic records after the removal of duplicates. Through a screening process outlined in Fig. 1, 37 papers were included in the review. A summary of all included papers is presented in Additional file 3. In the pages that follow, we provide a synthesis of the literature on IC in the healthcare sector, organized by research question. Each section offers a description of the findings and a commentary and critique. This is followed by a discussion of the state of the field and recommendations for future research.

### How is IC conceptualized and defined in healthcare organizations?

IC is defined in a variety of ways in the literature on healthcare, drawing on seminal works in the fields of accounting and strategic management. The most commonly used definitions describe IC as the sum or stock of knowledge that organizations use for value creation and competitive advantage [8, 9]. Several papers use the terms *intangible assets* and *intangible resources* instead of or interchangeably with IC [10–12], particularly those with a focus on IC valuation [13–15]; others prefer the terms *organizational knowledge* or *organizational intelligence* [6, 16]. Within the nursing literature, IC is defined as the stock of nursing knowledge available in an organization [17]. About 40 % of included papers do not provide a detailed definition for IC, choosing instead to describe IC as “intangible assets” or to briefly list categories or examples of IC (e.g., [18, 19]).

Despite some variation in terms, definitions, and underlying discipline, scholars accept the tripartite definition of IC developed in the broader literature in which IC consists of human capital, organizational capital, and relational capital. Human capital refers to the knowledge, skills and experiences owned and used by individuals [9, 20–24]. Structural capital (also called organizational capital) refers to institutionalized knowledge and codified experience stored in databases, procedures, routines and other organizational structures [9, 20–24]. Relational capital (also called social, stakeholder, or customer capital) refers to the knowledge embedded within, available through, and derived from networks of relationships internal and external to the organization [9, 20–24]. In other words, IC is accumulated and distributed in three different ways: through individuals, through organizational structures, processes, and systems, and through relationships and networks [9]. Human, structural, and relational capital can be considered separate constructs with their own nomological networks of antecedents, covariates, and consequences [25]; yet they are also interdependent and collectively responsible for shaping the stock of knowledge in an organization. Table 1 summarizes and synthesizes the content of each category across included papers, revealing a wide range of sources and examples of IC. These examples reflect those provided in the broader IC literature, suggesting that most IC sub-dimensions are consistent across industries. However, the unique characteristics of the healthcare industry – such as the highly politicized environment, information asymmetry between providers and patients, work processes and decisions that can mean life or death for patients, providers who have contractual (not employee) relationships with organizations and indirect payment for services by patients through third parties such as government programs and insurance companies – may contribute to significant differences in the relative importance, specific content, and antecedents and outcomes of these widely-accepted IC categories and sub-dimensions.

**Table 1:** Types of intellectual capital (IC) and healthcare examples

Type of IC	Definition	Examples
Human Capital	knowledge, skills and experiences owned and used by individuals	Professional competencies and judgment Specialized skills Context-specific knowledge Leadership and managerial skills Personal dispositions
Structural Capital	institutionalized knowledge and codified experience stored in databases, procedures, and the organizational culture	Vision, mission, values, strategic plan Programs, tools, and information systems Ways of working together Best practices Routines
Relational Capital	knowledge available through networks of relationships internal and external to the organization	Patient/caregiver views and experiences Nature of internal clinical-managerial relations Contracts/agreements and partnerships with other service providers or with government, research institutions, consultants, etc. Brand, image, and reputation in the community

The only major departures from the widely adopted IC categories of human, structural, and relational capital, can be found in studies by Wu and Hu [26], Chang et al. [27], and Habersam and Piber [10]. Wu and Hu [26] subsume relational capital under structural capital and include a separate category on “information capital”, which they define as the “information technology (IT) infrastructure and applications that support a hospital’s strategies, medical and administrative processes, clinical practices of medical professionals, and patient management processes” (p. 979). Other authors typically include IT technologies and systems under structural capital. In addition to human, structural and relational capital, Chang et al. [27] include “innovation capital”, which they define as having organizational structures and a culture that supports innovation. Drawing from their case studies of hospitals in Italy and Austria, Habersam and Piber [10] propose a fourth type of IC – connectivity capital – to capture examples of IC that span human, structural, and/or relational capital.

In terms of theoretical grounding, over a third of included papers (35 %) explicitly contextualize their roots in resource- and knowledge-based theories of the firm. The Resource-Based View (RBV) argues that a firm achieves sustainable competitive advantage from integrating its tangible and intangible resources, particularly those that are valuable, rare, inimitable or imperfectly imitable, and non-substitutable [2, 28]. Performance differences between organizations are a result of resource heterogeneity and the RBV is focused on the factors that cause these differences. The Knowledge-Based View (KBV) is an extension of the RBV that emphasizes knowledge and learning as the critical resources, and identifies the primary rationale for the firm as the creation and application of knowledge [8, 29]. Some of the papers also draw explicitly from human resources (HR) theories, which focus on investing in the education and career development of employees [23, 30]. The majority of papers (63 %) are not explicitly grounded in or informed by an overarching theory, choosing instead to use the three types of IC to frame their work. Reed et al. [31] argue that IC *is* a mid-range theory that is more amenable to hypothesis testing than the RBV and KBV because it more narrowly considers three types of intangible, knowledge-based resources, namely human, structural and relational capital.

However, all three perspectives – the RBV, the KBV, and IC – are typically only cited for two reasons: (1) to make the general point that intangible resources, such as knowledge, underpin organizational performance and competitive advantage and (2) to serve as conceptual frameworks to attempt to explain observed differences in performance [7, 19, 30, 32]. However, with the exception of three papers [21, 23, 26], these perspectives have not been used to develop testable hypotheses regarding which resources and knowledge are most significant, how they influence performance outcomes, what the conditions are that foster their development, or whether there is a real association between IC and performance. No papers test the link with environmental competitiveness. The weak use of theory in the literature on IC in healthcare may be exacerbated by the fact that IC is often defined in terms of the performance outcomes associated with it (i.e., competitive advantage). The RBV has similarly been criticized for circular reasoning, and for being impractical due to causal ambiguity as to the source of competitive advantage [33].

#### Commentary

Several conceptual and theoretical challenges were identified in the healthcare literature on IC, many of which reflect issues plaguing the broader IC field. First, a substantial number of papers failed to explicitly define IC (17/37 or 46 %) and often only refer to its sub-domains or use the term synonymously with “knowledge resources” or “intangible assets”, which are also rarely clearly defined. Papers that do not clearly define the key concept under study can contribute to conceptualizations of IC that are too broad or too narrow. Clear definitions support the operationalization of concepts for rigorous empirical testing and internal management use.

The most commonly used definitions of IC describe IC as the sum or stock of knowledge in an organization. However, some scholars raise concerns about equating IC with collective knowledge. Pettie and Guthrie [34] argue that some frequently evoked components of IC, such as reputation and stakeholder views, are byproducts of the management and use of IC (or lack thereof), but are not an inherent part of the organization’s IC (p. 158). Roos [35] similarly argues that company infrastructure and relationships are “not just knowledge” and raises the question of whether IC is best positioned within the narrowly focused KBV or the RBV (p. 152). These arguments imply that “intangible assets” is the broader term of which IC is a sub-domain focused only on knowledge. From this perspective, organizational brand and reputation, for example, would be considered intangible assets, but not IC.

Other scholars argue that intangible assets such as reputation are generated by “continuous, knowledge-based relationships” and therefore can and should be classified as IC [36]. Overall, most scholars do include a broad range of elements in their conceptualization of IC, including both those that are explicitly knowledge-based and those that are not [4, 10, 12, 19, 23, 37, 38]. This phenomenon may be attributed to the diverse ways in which the term has been used by theorists and practitioners in accounting, strategic management and information sciences. In accounting, the focus is on valuation and therefore IC is often used interchangeably with “intangible assets”. However, in strategic management and information sciences, the focus is on using IC to inform planning and decision-making, which requires unpacking IC content and identifying underlying knowledge.

The debate about whether IC is a subset of intangible assets or synonymous with intangible assets may be informed by deeper exploration of what we mean by “knowledge.” According to Plato, knowledge is defined as “justified true belief” [39]. The truth is based on facts and information. Beliefs are based on perception. Knowledge thus encompasses both reality and perception. An organization’s reputation or image, culture, and internal and external relationships are rooted in complex combinations of facts and perceptions. This argument suggests that it *is* appropriate to label these elements as “knowledge-based” and to classify them as IC. Regardless of the perspective selected, authors must be clear about which view they adopt and why.

Caution must also be exercised in how “capital” is defined, otherwise the application of an IC perspective becomes one of renaming each part of the organization in terms of its knowledge components, which limits the explanatory value of IC in research and practice. To qualify as IC, the knowledge in question must be sufficiently specific to enable its development and use as part of an organization’s strategy. Winter [40] argues that in order to qualify as capital, the knowledge in question must be transferable. He proposes several taxonomic dimensions with which transferability may be examined, including the extent to which the knowledge is articulable, teachable, and observable in use [40]. Another conceptual issue identified in the literature is that some applications of IC are narrowly focused on HR issues [12, 17, 30, 41–43]. This may occur, in part, because it is easier to conceptualize knowledge as a resource owned and stored by individuals, than as a resource that can be embedded in organizational structures and networks of relations. It is also relatively easier to measure (or estimate) the competencies and skills of individuals, using indicators like education level, years of experience, and hours of training, than it is to measure organization-level capabilities and relationships. Furthermore, data for indicators such as staffing levels, staff mix, and retention rates, are often accessible via HR databases in healthcare organizations, though they are not always systematically tracked, reported, and linked to performance outcomes.

The growing body of nursing IC literature, for example, adopts a narrow focus on human capital. IC indicators used in the nursing literature focus on nursing competence and skill, staffing, and recruitment and retention [17, 44]. Due to a lack of data, the authors were unable to measure ‘employer support for nursing continuing professional education’, which they conceptualized as a dimension of structural capital; they also did not discuss or operationalize relational capital [17, 45, 46]. Nursing IC papers thus make more of a contribution to the HR literature than to the IC literature [17, 30, 41, 44–47]. Moreover, conceptualizing IC at the individual and group level within single professions (e.g., nursing) offers limited insights for understanding and improving organizational performance. To advance the field we need to explore IC at the organizational level and across the myriad professional groups that interact frequently in the delivery of patient care.

Many of the conceptual hindrances outlined above can be linked to the need for stronger or more explicit use of theory in future studies. Although several authors refer to IC as a ‘theory’ [17, 31, 44–46], the IC framework does not meet formal criteria for theory development as outlined by Whetten [48]. Rather, the tripartite IC framework is a typology. Furthermore, while the RBV and KBV help contextualize IC as a concept and highlight its importance, they do not offer insights on how IC influences organizational performance and how IC can be developed and supported. Alternative theories are needed to guide hypothesis development. Several possibilities exist. A Transaction Cost Economics (TCE) perspective suggests that IC lowers transaction costs by enhancing the efficiency of information exchange and action. Although TCE has been widely criticized by proponents of the KBV, scholars have also argued that the perspectives are complementary and should be integrated and applied in research on IC [49]. An Attention-Based View (ABV) suggests that IC can help organizations capture and evaluate information, and help focus the attention of decision-makers on appropriate issues and answers, thereby influencing subsequent organizational moves [50]. An attention-based lens on IC considers more explicitly the role of management, and other behavioral influences, than TCE. Given that managers play a key role in resource selection, development, and orchestration [51], future research on IC must examine structure, strategy *and* agency. Resource dependence theory has particular relevance to relational capital, and can be used to explore differences in relative emphasis on, or investment in, relational capital across healthcare organizations, and potential links to perceived organizational legitimacy, power, autonomy, and dependence [52].

These diverse theories highlight several constructs and related variables that have yet to be explored in the IC literature on healthcare, including strategic position and fit; attention orientation or attention patterns; decision uncertainty, speed, and quality; and inter-personal or inter-organizational trust. Such variables may help explain how competitive advantage and enhanced organizational performance are achieved through IC. Applying multiple theoretical perspectives and exploring a wider range of variables and (more proximal) outcomes will enhance our understanding of the nature and value of IC, possibly resulting in the formation of a multi-level and multi-dimensional model of the different forms of IC and the pathways by which they influence performance.

### How is IC measured in healthcare organizations?

Among the empirical papers included in the review (59 %), the primary method used to study IC in healthcare organizations is cross-sectional questionnaires (*n* = 12). These questionnaires collect data on managerial, and in some cases clinical [21, 25, 27, 53, 54], perceptions of IC, most often in hospital settings though studies have also been conducted in long-term, hospice, and palliative care organizations [21, 25, 55]. Questionnaires measuring IC are typically divided into scales focused on human, structural, and relational capital respectively, or similar variants. Representative sample items from the few publicly available questionnaires are provided in Table 2. Only two quantitative studies did not use questionnaires. Lee et al. [37] measured IC disclosure on Australian hospital websites using an 85-item index/checklist. Erickson and Rothberg [56] analyzed existing data using organizational financial statements to calculate a variation of Tobin’s q to estimate the value of IC. Tobin’s q is a traditional method for measuring intangibles based on the ratio between an organization’s market value and book value.

**Table 2:** Sample survey items from studies of intellectual capital (IC) in healthcare

Type of IC	Dimension	Survey Item	Source
Human	Employee competence	My hospital is excellent in terms of medical & administrative personnel’s know-how	Wu & Hu, 2012 [26]
	Employee development	The centre devotes resources & effort to update & develop employee knowledge & skills	Lin et al., 2013 [54]
Structural	Culture	My hospital has a supportive culture that allows medical & administrative personnel to try things	Wu & Hu, 2012 [26]
	Access to information	Our hospital has a full range of handbooks & a complete knowledge management system for employees’ easy reference	Yang & Lin, 2009 [23]
	Information technology (IT)	My hospital has superior IT infrastructure to support hospital strategies	Wu & Hu, 2012 [26]
	External environment	My hospital possesses precise knowledge of competitor orientation	Wu & Hu, 2012 [26]
	Internal processes	Our hospital has an effective management process	Yang & Lin, 2009 [23]
Relational	Patient-centered	The centre prides itself on being patient-oriented	Lin et al., 2013 [54]
	Patient loyalty	Patients are highly loyal to the centre	Lin et al., 2013 [54]
	Partnerships	Employees have close interactions with partners	Lin et al., 2013 [54]
	Internal relations	Employees trust each other with open communication	Yang & Lin, 2009 [23]

All of the qualitative studies (*n* = 5) involved semi-structured interviews with managers in hospitals [7, 10, 12, 19] as well as other institutions such as long-term care homes, rehabilitation institutes, and community support service agencies [7, 38]. Only one study included clinical staff as part of the respondent sample [19]. These qualitative studies focused primarily on identifying and describing types of IC, and capturing perceptions of the relative importance of types of IC. The remaining empirical studies used mixed methods (*n* = 4), either a combination of administrative data and questionnaires [6, 17, 44] or interviews and questionnaires [53].

In general, the concept of IC resonated with participants as an important element of performance, though they were usually unfamiliar with the technical term [10–12, 19, 38]. Human capital was often viewed as the most important form of IC in healthcare organizations, followed by relational capital [4, 11, 12, 19, 38]. However, in one study of nurse supervisors’ perceptions of IC, structural and relational capital had higher importance ratings than human capital [27]. Most of the studies produced lengthy lists of examples of IC in healthcare organizations [4, 6, 10, 12, 19, 38]. An exception is Smith [7], who argued that the three most important intangible assets in healthcare are reputation, employee know-how, and organizational culture. Smith [7] refers to the RBV and limited previous literature to justify the importance of these three assets, but it is unclear whether his interviews with managers and employees in three healthcare organizations support his views or not. A discussion paper by Robinson [18] highlights three similar IC assets as sources of “long-term advantage”: consumer loyalty, clinical processes and improvement efforts, and culture and governance.

Only two studies used systematic approaches to identify and assess specific knowledge resources, as opposed to referring to generic categories of IC or supplying ad hoc examples [4, 6]. King & Zeithaml [6] conducted interviews and administered a questionnaire to 96 top and middle managers in eight community hospitals in North Carolina to identify and rank 30 ‘knowledge resources’ that provide competitive advantage. Seven categories of knowledge resources were identified in the following areas: managing human resources, clinical specialty capabilities, managing managed care, managing external stakeholders, information systems capabilities, facilitating innovative market extensions, and managing patient perceptions of care. The top three specific knowledge assets included knowledge, skills and experience in containing costs, succeeding in an environment of managed care, and maintaining a patient-friendly environment [51]. Similarly, Peng et al. [4] administered questionnaires to 30 hospital managers (9 of which also had clinical roles) in Taiwan to assess the importance of 59 IC assets identified via a literature review and expert consultation. The human capital category (consisting of seven assets) had the highest overall importance mean. Although the relational capital category (consisting of 20 assets) had the lowest overall importance mean, four of these assets had higher individual means than any other asset in the list of 59. The organizational capital category consisted of 32 assets organized into four groups: healthcare services and quality, marketing, strategic management, and IT.

There is consensus in the literature that most healthcare organizations lack a comprehensive and systematic approach to measuring IC [3, 4, 10–12, 19, 32, 38, 53]. Habersam & Piber [10] propose four levels of IC transparency: metric (can be quantified), literal (can be written down), intuitive (can be explained), and black box (cannot be explained). The literature on IC in healthcare is focused primarily on identifying metrics for IC (*metric*), and to some extent on codifying IC in organizational documents (*literal*). This emphasis is understandable given the challenges of identifying, measuring, and managing less transparent, or more tacit, forms of IC. The data sources most often mentioned for measuring or evaluating IC include patient satisfaction surveys, staff satisfaction surveys, administrative data, human resources data, performance data, and patient outcomes data [7, 10–12, 17, 19, 30, 37, 38, 44–47, 53, 55], which suggests that human and relational capital are measured more often than organizational capital [11]. Review of organizational documents, such as annual reports and websites, is also suggested as a source of information on IC [7, 37].

In the broader literature on IC, there is more focus on the financial valuation of IC than in the literature on healthcare. Financial valuation methods attribute a portion of an organization’s value first to those assets reported on the organization’s balance sheet. The remaining portion of “unexplained” value is attributed to the organization’s intangible assets. Four methods for intangible asset valuation in healthcare are described in the literature: the market approach (based on sales comparison), the cost approach (based on reproduction or replacement cost), the income approach (based on revenues, income, and cash flow), and the asset-based approach (based on asset accumulation) [13–15]. These papers do not apply the tripartite IC framework, but their examples of intangible assets align with the categories of human capital (e.g., the workforce), structural capital (e.g., electronic medical records) and relational capital (e.g., relationships with patients).

The extent to which accounting methods provide accurate and useful valuations of IC in healthcare organizations is unclear. No empirical studies were identified that apply such methods in a healthcare context. There are two potential reasons for this. First, intangible assets and IC represent largely separate scholarly traditions. This divide may explain the lack of application of accounting methods. Second, although accounting scholars and practitioners in other industries frequently use financial valuation methods to represent and compare IC across organizations and industries [57, 58], the majority of papers included in this review question whether IC can be captured, communicated, and managed based solely on financial indicators (exceptions include [13–15]) given the multifaceted purposes of healthcare, the range and complexity of treatments, settings and patient groups, and the challenges inherent in conceptualizing and measuring cost and quality of care. The complexity of performance measurement in healthcare has contributed to the widespread use of frameworks such as the Balanced Scorecard, which emphasize the importance of both financial and non-financial indicators [59, 60].

#### Commentary

In general, the methodological quality of empirical papers on IC in healthcare was poor, largely for two reasons: inadequate description of the methods and limited rigor and sophistication of the methods. In the quantitative studies (including four mixed methods studies with a questionnaire component), the surveys used were usually not provided [11, 16, 17, 21, 25, 27, 44, 53, 55, 61], and inadequate information was often presented on survey source and development, structure and length of the survey, types of respondents and/or psychometric properties [11, 21, 23, 25, 27, 53]. In one study, both the number and types of respondents were missing [55]. In other studies, the operationalization of key concepts was vague [16, 55], weak arguments were provided for hypotheses and associated conclusions on IC [54] and methods linked to particular findings were unclear [4]. Finally, several papers provided only partial, if any, discussion of study limitations [11, 16, 21, 54–56].

As Table 2 demonstrates, the quality of survey items that have been applied in IC research to date is weak. Best practices are often not followed in the wording of survey items [62]. For example, many survey items lack specificity and use jargon, vague language, unclear frames of reference and double barreled questions. It is not clear whether each scale reflects comprehensively the multi-faceted nature of the concepts of human, structural and relational capital. Because there is no consensus regarding the sub-dimensions of each type of IC, each study focuses on a different set of sub-dimensions in a seemingly ad hoc manner. We developed the “dimensions” column in Table 2 in an effort to organize and compare the diverse survey items across studies. Future research should draw from existing literature to develop a parsimonious, but still comprehensive, list of sub-dimensions relevant to healthcare for each type of IC – with each sub-dimension supported by theory and empirical evidence, where possible.

In the qualitative studies (including one mixed methods study with a qualitative component), the sampling method was rarely provided, two studies had participant sample sizes as low as 6 [12, 38], and two studies did not report the sample size or types of employees who participated [7, 53]. The interview questions used were pre-tested and explicitly outlined in only one study [6]; in the remaining studies, the interview questions were not provided, not pre-tested, and were often inadequately described in the body of the paper [7, 10, 53]. Furthermore, only one study conducted a verification procedure (member checking) to establish the credibility of interview results and author interpretations [6]. In one study, a central tenet of the proposed methodology, action research, does not appear to have been fulfilled, and the data which emerged from the multiple methods used were not adequately integrated or triangulated, thereby limiting contextualization of the results and potential implications of the findings [53]. Finally, like their quantitative counterparts, several qualitative studies also neglected to adequately identify and discuss study limitations [7, 10, 12, 19, 38, 53].

### What is known about the relationship between IC and the performance of healthcare organizations, and organizational efforts to improve performance through IC?

The link between IC and performance is critical as the value of IC lies in predicting and driving performance, not in IC itself. Yet, this is perhaps the least well developed area of the literature. A total of only eight empirical papers examined the relationship between IC and various organizational processes and outcomes [16, 21, 23, 25, 26, 44, 55, 61].

Three of the papers studied the link between IC and capacity for innovation in healthcare organizations. In general, relational capital played the strongest and most direct role in fostering innovative practices [16, 21], followed by organizational capital [25]. Three key factors mediated the relationship between IC and innovation: the knowledge sharing climate in the organization, knowledge sharing activities among employees, and employee attitudes towards and intention to share knowledge [21, 25].

Two other papers also examined IC as an antecedent, or input, variable. A nursing IC study found that nursing human capital (academic preparation, specialty certification, and experience) is associated with better quality of care, specifically fewer adverse events, and better nursing recruitment and retention [44]. A study of IC and knowledge management among hospitals in Taiwan found that information capital (IT infrastructure and applications) was less important as a “knowledge asset” than human and organizational capital [26]. Wu and Hu [26] also found that although IC shapes hospital financial and patient performance, this relationship is mediated by both process capabilities in internal and external management, and knowledge capabilities in knowledge acquisition, transfer, integration and application.

Three papers examined the role of IC as a mediating factor between proposed antecedents and organizational processes and performance. Al-Abrrow [61] found that the positive impact of transformational leadership on performance (operationalized as a focus on employees and a focus on patients) was explained by organizational learning and partially by IC. Bontis and Serenko [55] found that leadership positively affected structural capital as well as human capital (via feedback), and that structural capital influenced process execution. This study also revealed marginal increases in human, organizational and relational capital over a 5-year period (based on three data collection points). The authors attributed the improvements to organizational investments in staff training, IT, and cross-functional team meetings [55]. Yang and Lin [23] found that IC, particularly organizational capital, explained the impact of HR practices on performance (operationalized by perceptual measures of employee satisfaction, patient loyalty, turnover rate, and quality of care). Four HR practices contributed to the accumulation of IC, and shaped organizational performance, albeit in different ways: recruitment and selection and health and safety (via all three types of IC), performance appraisal (via organizational and relational capital), and training and development (via human capital). Compensation, the fifth HR practice, had no effect in explaining organizational performance.

Collectively, these eight studies suggest that IC does influence processes and performance in healthcare organizations. However, several other contextual variables shape the nature and extent of influence of IC, including HR practices, knowledge management activities, organizational climate, and leadership behaviours. Based on these results, the authors provide several recommendations for how to improve performance through IC. These include: (1) Developing strong networks among employees and encouraging teamwork; (2) Fostering an organizational context and culture that empowers employees, encourages learning, and facilitates knowledge sharing; (3) Codifying knowledge and experience in organizational databases and texts; (4) Measuring employee competencies and performance (human capital), as well as other forms of IC; and (5) Hiring, training and retaining the best employees [16, 21, 23, 25, 26, 44, 55, 61].

Other empirical and non-empirical papers included in this review also emphasize these recommendations [3, 4, 11, 17–19, 38, 41, 43, 47, 53, 63]. However, some unique suggestions also emerged. Erickson and Rothberg [56], for example, classified health sector organizations by their knowledge management activity (high or low based on Tobin’s q) and competitive intelligence (high or low based on self-reported secondary data). Their analysis revealed that hospitals have high competitive intelligence, but low knowledge management activity, and medical clinics have low competitive intelligence and low knowledge management activity. Erickson and Rothberg [56] thus argue that clinics do not necessarily require sophisticated knowledge management systems or competitive intelligence operations since most of the new knowledge they generate is tacit, and not proprietary or codifiable. For hospitals, on the other hand, they argue for the importance of protecting knowledge because explicit improvements to processes or products can be rapidly adopted by other hospitals. Other papers raised the importance of establishing a strong brand and reputation [7, 18].

#### Commentary

Findings regarding the association between IC and organizational processes and performance must be interpreted in light of the weak conceptual clarity and logic and poor methodological rigour of most of the studies. While many of these issues have been discussed in previous sections, two additional points are worth making here. First, in the nursing IC papers, commonly studied HR variables are merely reallocated to an IC framework [17] with results that mirror those from the larger literature on nursing HR [64]. It is therefore unclear what explanatory value the IC framework adds to the study of nursing HR. Second, some papers study relationships between concepts – such as IC and innovative capability or HR practices and human capital – that are deeply intertwined and potentially tautological. Results suggesting significant relationships among these concepts, and corresponding recommendations for practice, must therefore be interpreted with caution.

In the empirical literature on IC in healthcare, there is a stronger emphasis on measuring IC than on managing it. Numerous potential data sources and indicators are proposed, but only generic, high-level recommendations and examples are provided on how organizations use IC or try to improve it. Comprehensive and explicit recommendations are lacking for how to systematically manage IC. This may be due, in part, to the fact that IC has been examined in empirical studies as an antecedent and a mediator, but rarely as an outcome. Empirical evidence is thus lacking for which factors and processes contribute to IC. Additional research is required to understand what strategies managers can use to increase, deploy, improve and leverage IC in healthcare organizations, and to understand what factors influence the content, quality or value, and ease of transfer of IC within and across healthcare organizations.

## Discussion

Through its tripartite framework, IC encompasses a range of diverse organizational factors, including human resources, work routines, information technology, and stakeholder relationships among other intangible assets. Entire bodies of literature exist on each of these factors. The contribution to healthcare management of IC is that it unites these disparate fields of inquiry into a broader taxonomy of determinants of performance. Superior organizational performance is derived from a complex combination of organizational elements, including both tangible and intangible resources. Scholars argue that in our knowledge-based society, intangible resources are more likely than tangible resources to produce competitive advantage and superior performance [65, 66]. The concept and framework of IC offers a means to study the value of intangible resources and their potentially mutually enhancing interactions on organizational performance, and to determine how to manage systematically these resources *together* so that efforts across these diverse elements are optimized and synergistic.

The concept of IC also challenges researchers and decision-makers to unpack the capabilities of healthcare organizations to identify, understand and improve underlying intangible resources. To date, such efforts have been limited in the literature [6] with most scholars choosing to describe and measure generic aspects of IC rather than explore the fundamental knowledge resources that constitute IC in a particular industry or organization. This gap in research may be due to the fact that what constitutes IC in one industry or organization may not be the same for another due to differences in mandate, profile, history, and context. Therefore, to unpack and explore IC, an in-depth understanding of the context and organization is necessary. Comparative case study research focused on identifying and unpacking IC across multiple healthcare organizations may provide new insights into the nature of IC and its influence on performance.

Critics may ask how IC frameworks differ from or add value to existing performance measurement and management systems such as the Balanced Scorecard (BSC), which is already widely used in healthcare [59, 60]. The BSC focuses attention on balancing financial and non-financial indicators across four quadrants, such as: learning and growth, internal processes, patient outcomes, and financial outcomes [67]. There is overlap between the BSC and IC frameworks. Both are rooted in traditional theories of performance and competition in which contextual factors, structures, processes and behaviours interact to produce outcomes [68, 69]. Both also examine intangible resources. For example, the BSC’s “learning and growth” quadrant often encompasses human capital indicators, the “internal processes” quadrant encompasses elements of structural capital, and the “patient outcomes” quadrant encompasses one aspect of relational capital. However, the purpose of the BSC is to identify a parsimonious set of indicators linked to strategy to guide strategic management, while the purpose of an IC lens is to identify the most important intangible resources to organizational performance to guide the management of core competencies and capabilities [70]. The BSC and IC are thus complementary, not necessarily redundant [34, 70, 71]. In practice, an understanding of IC can help inform what should be on an organization’s BSC.

The results of this review demonstrate that although the literature on IC in healthcare is growing, it is not advanced. The conceptual, theoretical, and methodological limitations we identified create challenges for IC research and practical application in healthcare. To address these limitations and guide future research we offer several recommendations.

First, the scope for what constitutes IC must be more strictly defined. If an intangible asset cannot be described clearly enough to be transferrable, if it cannot be managed or protected enough to be sustained, and if it is challenging to put a value on it beyond saying it is of value, then it is likely too poorly defined to be considered IC. Strict guidelines for identifying IC will support the operationalization of the concept for rigorous empirical testing and internal management use.

Second, we recommend drawing from and combining multiple theoretical perspectives to develop more diverse and comprehensive propositions with which to empirically test IC not only as an antecedent and mediating factor, but also as an outcome. Potential theories to apply to IC include transaction cost economics, the attention-based view, and resource dependence, among others. Grounding future IC research in alternative theories beyond the use of the RBV and KBV may help inform our understanding of which resources and knowledge are most significant, how they influence performance outcomes, and what the conditions are that foster their development. The application and integration of alternative theoretical frameworks also enables comparison of the relative effectiveness of these theories in explaining the contribution of IC to organizational performance.

Third, we recommend moving beyond the use of cross-sectional questionnaires and high-level case studies focused on identifying types of IC and their relative importance. Some forms of IC may be inimitable due to protection by a patent, accumulated experience or cultural traits that are difficult to reproduce, and may thus pose challenges to researchers aiming to identify and describe them. The application of more diverse research methods may help in this regard. Administrative, human resources, and performance data can be used to complement questionnaire data. Social network analysis may also be used to examine relational capital, the results of which could complement the more general findings emerging from a questionnaire. Despite the potential benefits of integrating and triangulating data on IC from multiple sources, very few studies to date utilize a mixed methods research design. In-depth comparative case studies are also needed that unpack the content of IC assets and reveal how IC is used or not used. Given the challenges of such a ‘deep dive’, it may be necessary to scope studies so that they focus on particular departments or initiatives within or across organizations. Longitudinal research is also needed to understand the dynamic nature of IC and its relationship to performance over time. Only one study of IC in healthcare involved longitudinal data collection [55]. Although this study revealed minor changes in levels of IC over time, the reliance on high-level questionnaire data limits our understanding of how the specific content and nature of the organization’s IC resources may have evolved. Regardless of the methods used, advancement of the field demands the application of best practices in research design and reporting of methods and results.

Fourth, we recommend that scholars and practitioners break down what it means to “manage IC”. For each IC asset identified, we suggest exploring: What is the content of the asset? Where in the organization is the asset embedded? How is it currently used? Because many forms of IC are likely to span human, structural, and/or relational capital, rather than classifying assets explicitly under one of the three categories, it may be more useful to identify where in the organization the asset is embedded, thereby incorporating multiple sources at the human, structural and relational levels, if applicable. The absence of comprehensive and explicit recommendations for how to manage systematically IC means that we need research that applies a more nuanced and fine-grained lens to IC management. This involves attention to how IC is increased, deployed, improved, and leveraged, as well as to factors that influence the content, quality or value, and ease of transfer of IC.

Fifth, we recommend drawing from and building on bodies of literature related to IC, such as core competencies [72, 73], dynamic capabilities [74], knowledge management [75] and organizational learning [76]. Cross-fertilization of these concepts in the healthcare domain is lacking, and even in the general literature on IC there has been more focus on the relationships between IC and knowledge management, than on the relationships between IC and other concepts [77, 78]. There is much we can learn in healthcare from seminal works in bodies of literature related to IC as well as in the broader IC literature. Some seminal studies on IC outside of healthcare have a stronger foundation in existing literature and theory, more well-defined concepts and clear operationalization of concepts into measureable factors, more rigorous methods (or more complete reporting of methods and limitations), and more comprehensive and in-depth questionnaires on IC [9, 31]. That said, a previous literature review of IC reveals that many of the conceptual and theoretical weaknesses we have identified in this review are also common outside of the healthcare domain [79]. These two observations suggest that while we can learn from the broader IC literature to help advance research on IC in healthcare, the value of the IC construct may also have limited explanatory value in its current form. The latter point reinforces our recommendations to draw from alternative theories beyond the KBV and RBV, to integrate concepts and bodies of literature related to IC, and to conduct studies that involve a deeper ‘dive’ into IC content that go beyond the simple classification of resources under the tripartite IC framework.

Finally, the literature on IC in healthcare to date has focused on the organizational level. Although additional work is needed to advance knowledge of the nature and influence of IC at the organizational level, IC is also relevant to inter-organizational networks and to health system performance. Interventions aimed at improving efficiency and effectiveness system-wide, such as integrated care models, rely on the buy-in and alignment of multiple stakeholder groups, including diverse healthcare organizations and clinical providers. Furthermore, there is increasing interest in measuring health system performance based on the patient journey, as opposed to discrete episodes of care. Networks can facilitate the sharing and integration of data and resources, including IC, across organizations. This raises the question, “how can the IC of organizations and networks of providers across the full continuum of care be coordinated and leveraged to enhance the patient experience and the success of initiatives that span organizational boundaries?” An IC lens on health system performance and improvement may offer new insights on resource allocation and investment as well as mechanisms of change.

This review has some limitations. First, books and grey literature on IC in healthcare, such as organizational reports, were excluded and may offer further insight into the conceptualization and measurement of IC in the healthcare sector. Second, the review focused on literature situated in the healthcare domain and excluded the rich body of work on IC in other industries. A more direct and thorough comparison of papers on IC within and outside healthcare may enhance our understanding of how IC is operationalized and provide a more complete view of its evidence base (or lack thereof).

## Conclusion

With growing demands for innovation to improve quality of care, contain rising costs, and integrate services, more effective methods are needed to manage and measure IC both within and across healthcare organizations. This synthesis and critique of the literature on IC in healthcare has implications for both researchers and leaders. Given that the application of an IC framework to healthcare organizations is relatively new and underdeveloped, there are ample opportunities for researchers to contribute to scholarship in this area of inquiry. The review identified potential pitfalls to avoid and provides several recommendations to guide future research. For healthcare leaders and decision-makers, the review offers an introduction to the concept of IC, its potential value to healthcare management and delivery, a summary of the evidence to date on its link to organizational processes and performance, and high-level suggestions for how to build IC in healthcare organizations.

## Additional files

Additional file 1: **A bibliometric analysis of research on intellectual capital in healthcare.** (PDF 239 kb)
Additional file 2: **PRISMA Checklist.**(DOC 63 kb)
Additional file 3: **Overview of peer-reviewed published papers on intellectual capital in healthcare (*****n*** **=** **37).** (PDF 327 kb)

## Abbreviations

ABV: Attention-based view
BSC: Balanced Scorecard
HR: Human resources
IC: Intellectual capital
PRISMA: Preferred Reporting Items for Systematic Reviews and Meta-Analyses
RBV: Resource-based view
TCE: Transaction cost economics
KBV: Knowledge-based view
