# Supplementary material for: Intellectual capital in the healthcare sector: a systematic review and critique of the literature
Source: BMC Health Serv Res. 2015 Dec 15;15:556. doi: 10.1186/s12913-015-1234-0 (PMC4678586; doi:10.1186/s12913-015-1234-0)
Supplement: Additional file 1: — A bibliometric analysis of research on intellectual capital in healthcare. (PDF 239 kb) [file 12913_2015_1234_MOESM1_ESM.pdf]

Additional File 1

A bibliometric analysis of research on intellectual capital in healthcare

Methods

Bibliometrics is the application of quantitative analysis and statistics to publications based on information such as authors, journals, and keywords to identify meaningful structures and patterns. We conducted a bibliometric analysis to answer the following research question: How has the research on IC in healthcare organizations evolved (or not) over time in terms of foci, contributors, impact and publishing outlets?

We collected general citation information, including author(s), author affiliation(s), journal, year of publication, number of citations, keywords, and topic(s) covered. On multi-author papers, we gave each author equal credit for the paper. If the authors of a paper shared the same affiliation, we counted the paper only once for that institution. However, if the authors of a paper had different affiliations, we counted the publication more than once – all institutions got equal credit for it. When an author had multiple affiliations, we used only the first-listed institution. The number of citations for each paper was recorded using Google Scholar, which captures a more broad set of publications than other databases that offer citation data, such as Web of Science and Scopus. Finally, the assignment of topics to publications was done manually, based on the full text of the papers, using the following broad categories: (1) identifying, describing, and ranking IC; (2) measuring and managing IC; (3) accounting methods for valuing IC; (4) linkage between IC and organizational processes and performance and (5) IC, technology and information systems. These categories were developed iteratively during the process of reviewing and synthesizing the included papers. Although some papers span multiple

categories, we assigned a primary category based on the main focus of the paper. Frequency tables were used to analyze the bibliometric data and identify meaningful patterns.

Results

Papers on IC in healthcare emerged in 1997-1998, with three publications, but did not peak until 2007-2008 when nine papers were published, and 2012-2013 when 11 papers were published. In general, the literature on IC in healthcare is growing, though growth is punctuated by periodic declines (Figure 1).

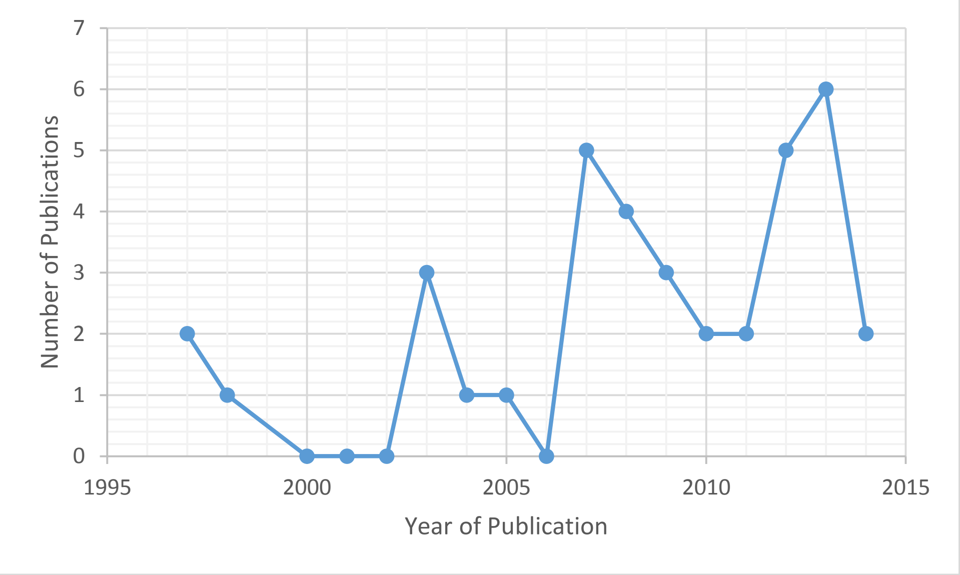

Figure 1. Number of Publications on Intellectual Capital in Healthcare 1997-2014

The 37 papers included in the review spanned five topic areas, as outlined below.

- *Topic 1: identifying, describing, and ranking IC (11 papers)* (Carclucci & Schiuma, 2012; Chang et al., 2014; Habersam & Piber, 2003; King & Zeithaml, 2003; Kong, 2008; Peng et al., 2007; Robinson, 1998; Smith, 2008; Sillanpaa et al., 2010; Zigan et al., 2008; Zigan et al., 2009);

- *Topic 2: measuring and managing IC (10 papers)* (Corso, 2007; Covell & Sidani, 2013b; Erickson & Rothberg, 2013; Grantham et al., 1997; Hall, 2003; Lee et al., 2007; Moody, 2004; Price, 2013; Wall, 2005; Weston et al., 2007);
- *Topic 3: linkage between IC and organizational processes and performance (10 papers)* (Al-Abrrow, 2014; Bontis & Serenko, 2009; Covell, 2008; Covell & Sidani, 2012; Covell & Sidani, 2013a; Mura et al., 2012, Radaelli et al., 2011; Santos-Rodrigues et al., 2012; Wu & Hu, 2012; Yang & Lin);
- *Topic 4: accounting methods for valuing IC (3 papers)* (Reilly & Rabe, 1997; Reilly, 2010; Reilly 2012); and
- *Topic 5: IC, technology and information systems (3 papers)* (Lin et al., 2013; Poe, 2011; Simpson, 2007).

Ten of the papers distributed amongst the above categories are focused on the field of nursing IC (Covell, 2008, Covell & Sidani, 2012; Covell & Sidani, 2013a; Covell & Sidani, 2013b; Hall, 2003; Moody, 2004; Poe, 2011; Price, 2013; Simpson, 2007; Weston, 2007).

The keywords selected by authors also reveal important information regarding the foci of papers and how IC is conceptualized. Eight papers (8/36 or 22%) did not report keywords. Twenty papers (19/36 or 54%) used IC as a keyword. Papers that did not use IC as a keyword, used alternative terms such as human capital (common in the nursing IC literature), intangible assets, knowledge management, asset management, or information technology. Aside from IC, popular keywords included (in order of decreasing frequency): organizational performance (and variants such as performance measurement/measures and performance/service improvement); intangible assets; and knowledge management.

The majority of contributing authors (52/65 or 85%) have only published one paper on IC in healthcare, though it is possible that some of these scholars have published on IC in non-healthcare settings. Ten authors (15%) have published between two and four papers on IC in healthcare. The author with the most publications is Christine Covell whose work focuses on nursing IC.

Forty institutions were represented by the authors with the majority (33/38 or 87%) linked to only one paper on IC in healthcare. The institutions included 32 universities, four consulting firms, and four healthcare organizations. Seven institutions were represented by two or three papers. These include three universities in Canada, one university in the UK, one university in Taiwan, and a consulting firm in the US. The institutions are distributed across eleven countries: the US (14), Canada (10), Taiwan (6), the UK (4), Finland (3), Australia (2), Italy (2), Austria (1), Iraq (1), Ireland (1), and Portugal (1).

Included papers were published in 29 journals. We categorized these journals to determine where IC papers on healthcare are typically published. The categories, and associated number of papers, include: Nursing journals (10 papers), Business, Management or Public Administration journals (9 papers), Knowledge Management or Intellectual Capital journals (5 papers), Accounting or Finance journals (4 papers), Healthcare journals (4 papers), and Information Technology journals (2 papers). Journals with multiple IC publications include *Journal of Advanced Nursing* (3 papers), *Nursing Administration Quarterly* (3 papers), *Journal of Health Care Finance* (3 papers), and *Health Care Management Review* (2 papers). The majority of journals (17/29 or 59%) have no reported impact factor. Eight journals (28%) have an impact factor above one and three journals (10%) have an impact factor above two. The three journals with the highest impact factors, each of which published one paper on IC in healthcare,

are *Health Affairs*, the *Strategic Management Journal* and the *International Journal of Management Reviews*.

Over a third of papers on IC in healthcare have received between zero and five citations each (13/37). Ten papers have received over 30 citations, five over 50, and two over 100. The two papers with over 100 citations, suggesting a seminal contribution to the literature, include "Measuring Organizational Knowledge: A Conceptual and Methodological Framework" (King & Zeithaml, 2003) and "The Development of Strategic Management in the Non-Profit Context: Intellectual Capital in Social Service Non-Profit Organizations" (Kong, 2008).

Commentary

The bibliometric analysis suggests that IC in healthcare is a field of research and inquiry still in its early stages of development. The number of papers identified is relatively low (n=37) and although there is an upward trend in the number of papers published between 1997 and 2014, this growth is punctuated by periodic declines. Furthermore, the literature to date, including empirical and non-empirical studies, is primarily descriptive in nature and published in low impact journals with a relatively low level of citation overall. Finally, few scholars are dedicated to conducting research on IC in healthcare (i.e., have published more than one paper on the topic), which suggests that expertise on IC in the healthcare industry is weak and distributed. These findings highlight weaknesses in the scholarship on IC in healthcare and the need for a critical review of the literature, but also indicate an opportunity for researchers and practitioners to contribute to the advancement of the field.
