# Supplementary material for: Intellectual capital in the healthcare sector: a systematic review and critique of the literature
Source: BMC Health Serv Res. 2015 Dec 15;15:556. doi: 10.1186/s12913-015-1234-0 (PMC4678586; doi:10.1186/s12913-015-1234-0)
Supplement: Additional file 3: — Overview of peer-reviewed published papers on intellectual capital in healthcare (n = 37). (PDF 327 kb) [file 12913_2015_1234_MOESM3_ESM.pdf]

Additional File 3: Overview of peer-reviewed published papers on intellectual capital (IC) in healthcare (n=37)

Author(s)	Title	Purpose	IC Definition	Theoretical Framework	Methods	Results or Key Points
Al-Abrrow, 2014*	Transformational Leadership and Organisational Performance in the Public Healthcare Sector: The Role of Organisational Learning and Intellectual Capital	To investigate the influence of transformational leadership style on organizational performance depending on both organizational1 learning and IC	Defined as valuable assets that are a source of competitive advantage and value creation; consists of human, structural, and customer/relational capital	Transformational leadership (authors view leadership style as an antecedent to IC)	Questionnaire to gather individual-level quantitative data from 189 employees from 10 hospitals in the UAE (96% RR)	Transformational leadership has a significant positive effect on organizational performance (defined as a patient focus and an employee focus), and this relationship was moderated by both organizational learning (fully) and IC (partially). A significant positive relationship was found between organizational learning, IC and organizational performance.
Bontis & Serenko, 2009*	Longitudinal knowledge 1strategising in a long-term healthcare organisation	To identify the inputs and outputs of effective knowledge management over time in a non-profit healthcare setting	No explicit definition provided; but refer to three components of human, structural and relational capital	No explicit underlying theory; draw from literature on IC, leadership, and various HR concepts	Questionnaire administered three times (2001, 2003, 2005) in a long-term healthcare organization in ON, Canada. No information provided on number or type of respondents (average 75% RR)	The more committed employees are, the more likely they are to generate knowledge for the organization. Managerial leadership is the key antecedent factor for knowledge management; it affects retention of employees, value alignment, structural capital and perception of supervisor feedback. It is important to combine human resources (HR) practices with knowledge management activities.
Carlucci & Schiuma, 2012*	Evaluating organizational climate through IC lens: the case of a public hospital	To examine how IC provides a useful frame for analyzing intangible components of organizational climate and for management initiatives	Broadly defined as the "intangible resources embedded in organizations"; consists of human, structural, social and stakeholder capital	No explicit underlying theory; uses the Knoware Tree as a conceptual framework (Schiuma et al., 2005)	Action research methodology with a public hospital in Italy, involving document analysis, observation, interviews, focus groups, and a survey (560 employees, 51%RR)	IC components identified as weak: knowledge of hospital's mission, vision, strategic plan; teamwork; physical workspace; organizational rewards; management support; training and employee competencies; info sharing and dissemination at organizational level; and conflict management. IC strengths include organizational identity, empowerment and customer-centeredness.

Chang et al., 2014*	Perceptions of intellectual capital held by the supervisors of nursing divisions in hospitals in Taiwan	To explore how nursing supervisors perceive IC and identify the relative importance of IC factors	"knowledge, information, intellectual property, experience or management system that can bring sustained competitiveness to an organisation" (Stewart, 1997); categorized as human capital, structural capital, relational capital and innovation capital. HC: sum of employees' competencies, interactions, responsiveness and attitudes towards work. RC: relationships with in-patients, out-patients, and supervisors. SC: organizational learning, operating processes, and information systems. Innovation capital: structures (e.g., investment, incentives) and culture that support innovation	No explicit underlying theory; draws from literature on IC	Fuzzy Delphi method using a questionnaire completed by nursing division supervisors at public teaching hospitals in Taiwan (# of respondents and response rate not reported)	The average importance ratings were highest for structural capital (6.49) followed by relational capital (6.41), human capital (6.16), and innovation capital (6.04). The four sub-dimensions with the highest ratings were organizational learning (6.74) (structural capital), operating processes (6.68) (structural capital), relationships with inpatients (6.52) (relational capital), and employees' attitude towards work (6.52) (human capital).
Corso 2007	Rethinking traditional methods for measuring intellectual capital	To outline transitional measures, current measures, and future directions for measuring IC	No explicit definition provided; authors state that term IC coined as replacement for the accounting term "intangible assets"	No explicit underlying theory; draws from literature on IC	Discussion paper	Traditional measures (Goodwill & Tobin's Q) inadequate for capturing complexity of value of IC to organizations. The balanced scorecard (and other similar systems) focus on the human, knowledge, learning and info elements in organizations. Standardization of IC measures across organizations likely unattainable due to diversity.
Covell, 2008	The middle-range theory of nursing intellectual capital	To report on the development of the middle-range theory of nursing IC	"organizational knowledge that is translated into business performance" (Bontis, 1999). Nursing IC is "nursing knowledge that is translated into nursing and organizational performance"	No explicit underlying theory; draws from literature on IC and uses IC as a theoretical framework	Discussion paper	Proposes that nurse staffing and employer support for continuing professional development are associated with nursing human capital; nursing human capital is associated with patient and organizational outcomes; and nursing structural capital is associated with patient outcomes.

Covell & Sidani, 2012*	Nursing intellectual capital theory: operationalization and empirical validation of concepts	To present the operationalization of concepts in the nursing IC theory and the results of a methodological study aimed at empirically validating the concepts	No explicit definition of IC. Nursing IC defined as "the stocks of nursing knowledge available in an organization"	No explicit underlying theory; draws from literature on IC and uses IC as a theoretical framework	Administrative databases and questionnaires with unit managers from 91 inpatient units at 6 acute care hospitals in two Canadian provinces	Nursing human capital concept validated with three indicators: hours provided to patients per day, skill mix, and RN-to-patient ratio. Problems identified with operationalization of nursing structural capital and employer support for continuing professional education due to data availability.
Covell & Sidani, 2013*	Nursing intellectual capital theory: testing selected propositions	To test selected propositions of the middle-range theory of nursing IC	No explicit definition of IC. Nursing IC defined as "the stocks of nursing knowledge available in an organization"	No explicit underlying theory; draws from literature on IC and uses IC as a theoretical framework	Administrative databases and questionnaires with unit managers from 91 inpatient units at 6 acute care hospitals in two Canadian provinces	Nursing human capital influences quality of patient care and nursing recruitment and retention. Propositions associated with nursing structural capital not tested.
Covell & Sidani, 2013	Nursing Intellectual Capital Theory: Implications for Research and Practice	To review the nursing IC theory and discuss its implications for research and practice	"combination of collective knowledge of individuals and structures in an organization" that is translated into business performance (Business Dictionary, 2013; Bontis, 1999); consists of human, structural and relational capital. Nursing IC is "the stocks of nursing knowledge available within a healthcare organization"	No explicit underlying theory; draws from literature on IC and uses IC as a theoretical framework	Discussion paper	Summarizes content of previous papers by Covell (concepts, propositions, empirical evidence to date). Relational capital not translated for nursing context.
Erickson & Rothberg, 2013*	A Strategic Approach to Knowledge Development & Protection	To apply the Strategic Protection Factors Framework to healthcare industries	IC not explicitly defined aside from referring to its focus on measuring and assessing knowledge asset stocks, and its three components: human, structural and relational capital	No explicit underlying theory; draws from literature on knowledge management, IC, and competitive intelligence	Two variations of Tobin's Q using existing financial data, and self-report on maturity of firm's competitive intelligence (secondary data), for 506 firms including hospitals and clinics	Raises question of extent to which knowledge should be developed and shared versus protected. Strategic Protection Factors Framework consists of 4 archetypes based on knowledge management activity (high or low) and competitive intelligence (high or low): Cold War, Glass House, 800-Pound Gorilla, and Brilliance. Hospitals classified as glass houses with high competitive intelligence but low knowledge management.

						Clinics classified as brilliance with low competitive intelligence and low knowledge management. Decisions about knowledge development and protection are highly context-specific and should be explicit strategic choices based on an understanding of the competitive environment.
Grantham et al. 1997	A Framework for the Management of Intellectual Capital in the Health Care Industry	To propose a theoretical model for managing IC	"the intellectual material that has been formalized, captured, and leveraged to produce a higher-valued asset" (Stewart, 1994); "information, intelligence, knowledge and wisdom"; consists of human, structural and customer capital	No explicit underlying theory; limited papers on IC cited	Discussion paper	Propose that an organizational design model based on info flows be used as a diagnostic tool and design template for managing IC. Proposed model of info flow consists of 6 organizational dimensions: growth (scale/rate of), focus (efficiency to remove waste), autonomy (rate of innovation), order (efficiency in terms of sequence), identity (uniqueness), and interaction patterns (internal and external connectedness). Proposes use of computer simulation and pie diagrams to visualize IC (not well-explained)
Habersam & Piber, 2003*	Exploring intellectual capital in hospitals: Two qualitative case studies in Italy & Austria	To explore the relevance and awareness of IC in hospitals as well as existing practices and metrics used	Explicit definition not provided. IC described as intangible assets and as consisting of human, structural and relational capital	No explicit underlying theory; refers to the resource-based view in passing only	Semi-structured interviews with managers in an Italian and Austrian hospital (n=14 total)	There is an awareness of IC in hospitals and its measurement and management judged as highly relevant. Examples of human, structural and relational capital are provided. Propose concept of connectivity capital to help identify links among the three types of IC. Framework of IC transparency: metric capital, literal capital, intuitive capital, black box capital. In two cases, one organization has a more collective approach to IC conceptualization and management (IC is codified), and one was more focused on the individual level (structural versus human capital).
Hall 2003	Nursing intellectual capital: A theoretical approach for	To present a framework for examining productivity in nursing	"human capital combined with structural labor inputs" and "a combination of individual characteristics,	Human capital theory; implicitly draws from knowledge-based view	Pilot test with sample of community agencies in Ontario [methods not described]	The framework consists of individual nurse characteristics (education, experience, career planning & development, autonomy, organizational trust, organizational

	analyzing nursing productivity	that incorporates knowledge and skill, and uses preliminary data from a pilot study conducted in Canada to verify the dimensions of the model	education, experience, and attitudes" (Hudson, 1993)	of the firm; limited IC papers referenced		commitment, job satisfaction) and nursing productivity (nursing costs, turnover, absenteeism, replacement costs, orientation costs, education costs). The model also includes organizational support for knowledge work, and, as outcomes, nursing error related to patient safety and patient satisfaction with nursing care.
King & Zeithaml, 2003*	Measuring organizational knowledge: A conceptual and methodological framework	To present a framework and research protocol to identify organizational knowledge resources within industries	Do not use the term IC. Use the term "organizational knowledge", but their conceptualization of this term aligns with IC (examples of human, structural, relational capital)	Resource-based view of the firm and knowledge-based view of the firm	Semi-structured interviews with CEOs in the hospital (n=8) and textile (n=9) industries as well as questionnaires assessing the identified knowledge resources with top and middle managers (n=224)	Most important organizational knowledge resources or competencies: clinical capability of physicians, negotiating contracts, patient-friendly environment, tracking patient satisfaction, total quality management, and outpatient surgery. Least important: external relations, info systems, innovative partners, and tracking individual knowledge and skills. Resources identified fall into IC framework (though authors do not use this framework). Cannot generate inventory of generic knowledge resources applicable across industries. Some knowledge resources are not accessible using quantitative measures.
Kong, 2008	The development of strategic management in the non-profit context: Intellectual capital in social service non-profit organizations	To compare various strategic management concepts and tools, and to argue for the applicability of IC to social service non-profit organizations	"the possession of knowledge, applied experience, organizational technology, customer relationships and professional skills that provide…a competitive edge in the market" (Edvinsson & Malone, 1997); consists of human, structural and relational capital	Resource-based view of the firm; & knowledge-based view of the firm are discussed, including limitations	Discussion paper	Authors argue that IC is a strategic management framework that is appropriate for use in the social care sector, and is superior to the balanced scorecard, SWOT analysis, and resource-based and knowledge-based views.
Lee et al., 2007*	Is communicating intellectual capital information via the internet viable?	To examine the nature and extent of IC information Australian hospitals	No explicit definition provided aside from reference to IC's role in driving value creation; IC	No explicit underlying theory	Research team review of 128 Australian hospital websites in 2005 using an 85-item index developed by the authors in	IC disclosure index consists of 6 categories: human resources capital, patient capital, IT capital, process capital, innovation capital and strategic capital statements. Highest

	Case of Australian private and public hospitals	disclose to their stakeholders online and to examine whether 4 hospital characteristics influence disclosure	framework used based on Hermansson et al. (2004): education, development and training perspective, work environment perspective, and patient perspective		consultation with hospital representatives	rates of disclosure for HR and IT and lowest for innovation and process. Incidence rate of disclosure of IC information among hospitals is high, but the extent of IC disclosure is low (1/3 of 85 items). Amount of information disclosed varied by state location, private versus public hospitals, specialized versus general operations, and city or regional location. Hospital status is a network was not associated with disclosure.
Lin et al., 2013*	Physicians' participation in practice of knowledge management systems	To investigate physician satisfaction with knowledge management systems, and the relationship between satisfaction and IC	"Knowledge that is employed for competitive advantage" (Youndt et al., 2004); consists of human, structural and relational capital.	-Task Technology Fit Theory<br>-Draws from literature on IC and knowledge management	Questionnaire with 181 physicians in 13 hospitals (15% RR) in Taiwan	System quality, information quality, and service quality all have significant correlations with physician satisfaction with knowledge management systems. User satisfaction is significantly correlated with overall IC as well as HC, SC, and RC. The higher the user satisfaction, the better the IC.
Moody, 2004	Nurse productivity measures for the 21st century	To outline multiple views of nursing productivity: economic interdisciplinary cost ratios, knowledge work classification systems, and IC models	IC not explicitly defined. Use term "human IC" instead, referring to "workers' knowledge, competencies and skills"	No explicit underlying theory; draws from literature on nursing productivity (limited references on IC)	Discussion paper	Future examinations of nursing productivity must consider interdisciplinary productivity measurement, nurses knowledge classification systems, patient-nurse relationship, and human IC.
Mura et al., 2012*	Intellectual capital and innovative work behaviour: Opening the black box	To introduce and empirically test a theoretical model linking IC to employee innovative work behaviour	"the sum of all knowledge that organizations utilize for competitive advantage (Subramaniam & Youndt, 2005); consists of human, organizational and social capital. HC: set of knowledge owned and used by individuals. OC: codified knowledge present in procedures, guidelines,	- Knowledge-based view of the firm<br>- Theory of planned behaviour (to explore intention to share knowledge)	Questionnaire with 135 clinical staff (65% RR) from three hospice and palliative care organizations in Italy	Relationship between IC and innovative work behavior is mediated by knowledge sharing. Structural social capital directly linked to innovative behavior; human capital, affective social capital, and organizational capital are not. Increase in IC does not directly translate into knowledge sharing; mediated by practitioner willingness.

			databases, and manuals. SC: interactive knowledge that individuals have access to through social networks (ibid).			
Peng et al., 2007*	Intellectual capital and performance indicators: Taiwanese healthcare sector	To investigate how hospitals view the importance of IC and performance in the healthcare sector	"valuable, intangible and inimitable resources for value creation of a firm" (also refer to definitions by Roos & Roos, 1997, Roos et al 2005, and Nahapiet & Ghoshal 1998; consists of explicit/tacit and individual/social knowledge as well as other classifications such as human, structural and relational	No explicit underling theory; draws from the literature on IC and the balanced scorecard, and uses IC as a theoretical framework	(1) Review of literature and practice to identify elements of IC and indicators; (2) Expert review of list (n=5); and (3) Pilot test questionnaire with 30 managers (38% RR) in Taiwan. Also use the IC Navigator and Effector Plot to examine resource transformation between various types of IC.	Human capital ranked as most important, followed by organizational capital (service and quality, then marketing, then strategic, then IT). Although relational capital ranked last (based on overall mean), items in this section had the highest importance values. Performance measures domains in order of importance: operation efficiency, cost control, income and growth, clinical and medical quality, productivity. Human capital is a value source but gets less investment, organizational capital has more influence but is a value sink.
Poe, 2011	Building nursing intellectual capital for safe use of information technology	To conduct a systematic literature review to answer the question "What are the best practices to build nursing IC for use of IT for safe clinical care?"	No explicit definition provided. Nursing IC defined as "nurses' knowledge, skill and experience"	No explicit underlying theory; draws from literature on nursing IC and IT	Systematic literature review of papers published between 1998-2008 (n=55)	55 articles identified (18 empirical). Themes include nursing informatics support requirements, patient safety threats and IT, and nursing and informatics competencies.
Price, 2013	Understanding g nursing 'nous' in the context of service improvements	To describe the concept of 'nous' in service improvement and how the concept may be integrated into appraisals and evaluations	"hidden assets if the company, omitted in its balance sheets" (Wachowiak, 2005); consists of human, structural and relational capital	No explicit underlying theory; draws from literature on IC (limited) and nursing (limited)	Discussion paper	Nous refers to the combination of skills used to produce results. The authors argue that a better understanding is needed of what nurses do, what skills they use, and how they evaluate their work. Authors argue that structural and relational capital are of more value to service improvement than human capital.
Radaelli et al., 2011*	Intellectual capital and knowledge sharing: the	To explore the effect of IC on practitioners' knowledge-sharing	"the sum of all knowledge that organizations utilize for competitive advantage	No explicit underlying theory; draws from literature on IC and	Questionnaire with 155 healthcare professionals in three	Organizational and social capital are positively and significantly related to knowledge sharing behaviour; human capital

	mediating role of organizational knowledge-sharing climate	behavior, and propose organizational knowledge-sharing climate as a mediator	(Subramaniam & Youndt, 2005); consists of human, organizational and social capital. HC: set of knowledge owned and used by individuals. OC: codified knowledge present in procedures, guidelines, databases, and manuals. SC: interactive knowledge that individuals have access to through social networks (ibid).	knowledge management	hospice and palliative care organizations (66% RR) in Italy	is not. All three types of IC positively affect knowledge sharing climate. Knowledge-sharing climate positively affects knowledge-sharing behaviour. Knowledge-sharing climate fully mediates relationship between organizational capital and knowledge sharing; same for social capital. Authors argue that results confirm distinction between IC and knowledge management.
Reilly & Rabe, 1997	The valuation of health care intangible assets	To outline methods for the identification, valuation, and remaining useful life analysis of the typical intangible assets of physician practices (and other health care organizations)	Do not use term IC, use intangible assets. Intangible assets must have a specific bundle of legal rights associated with their existence, generate measureable economic benefit, and enhance the value of other assets with which they are associated.	No explicit underlying theory	Discussion paper	Intangible assets in healthcare: technology-related, patient-related, contract-related, data processing related, human capital related, marketing related, location related, and goodwill related. Market approach valuation is based on sales comparison. Cost approach is based on reproduction or replacement cost. Income approach is based on revenues, income and cash flow.
Reilly, 2010	Intangible asset valuation, damages, and transfer price analyses in the health care industry	To summarize the intangible assets common in the health care industry, and to describe the different types of intangible asset analyses and reasons for valuation	Does not use term IC, uses intangible assets. No explicit definition provided aside from statement that the value of intangible assets comes from the legal rights, intellectual content and expected economic benefits associated with them.	No explicit underlying theory	Discussion paper	Summarizes types of intangible assets in healthcare (same as in other Reilly papers); types of analyses: valuation, transfer price, lifting, damages, and fairness; and 10 reasons to analyze intangible assets (e.g., taxation, management information and strategic planning, litigation claims, bankruptcy, financing transactions, corporate governance and regulatory compliance, etc.)
Reilly, 2012	Cost approach of health care entity intangible asset valuation	To discuss common types of intangible assets in healthcare, accepted valuation	Does not use term IC, uses intangible assets. No explicit definition provided for intangible assets.	No explicit underlying theory	Discussion paper	Examples of healthcare intangible assets: patient relationships, EMR software, workforce, procedures systems manuals, employment agreements, service agreements, purchase agreements, license

		approaches, and the cost approach.				agreements, joint development/venture agreements, goodwill. Article provides details of how analysts should complete valuation assessments based on three methods.
Robinson, 1998	Financial capital and intellectual capital in physician practice management	To analyze the role of capital in the success of physician practice management firms	No explicit definition, but highlights three key sources of IC: org culture and governance, clinical processes, and brand-name recognition. Human capital mentioned in passing only (physician leaders).	No explicit underlying theory a resource-based view is implicit)	Discussion paper	Long-term competitive advantage will be derived from difficult-to-replicate innovations in 3 areas: organizational culture and governance, clinical processes, and brand-name recognition. The challenge is to translate financial assets into these intellectual assets.
Santos-Rodriguez et al., 2012*	Intellectual capital and innovation: A case study of a public healthcare organization in Europe	To investigate the influence of IC on capability for innovation	IC not explicitly defined aside from referring to its three components: human, structural and relational capital. Defined "organizational intelligence" as "represented by information and knowledge systems, the skills and abilities of employees, the quality of production processes, and customer service".	No explicit underlying theory; draws from literature on IC	Questionnaire with 68 service directors (95% RR) of a hospital in Portugal	Human capital: formation of knowledge, innovation attitude, creativity, incentives to innovate. Structural capital: culture, trust, organizational support for creation and development of knowledge. Relational capital: networks and alliances and clients. Human capital is associated (weakly) with innovation creation and incentives to innovate. Structural capital is associated with innovation adoption, but not innovation creation. Relational capital is associated with innovation creation and adoption.
Simpson, 2007	Building nursing intellectual capital for the information age	To discuss patient-centered IT tools	Defined in terms of its contents: human, structural and relational capital.	No explicit underlying theory though a knowledge-based view is implicit; draws from literature on nursing (no IC papers referenced)	Discussion paper	Authors argue that it is less about how many nurses we have and more about what they know. Technology (structural IC) can help support and extend human capital. Eight types of knowledge-based tools are outlined.
Smith, 2008*	Resource-based view of the firm: Measures of reputation among health service-sector businesses	To explore the significance of reputation as a strategic asset in health service organizations	Does not use term IC, uses term "intangible assets," but conceptualization implicitly aligns with IC (examples of human, structural and relational capital). Intangible	Resource-based view of the firm	Interviews of upper to middle management, and comments from convenience samples of employees in three organizations: Baptist Homes, University of Pittsburgh Medical Center, and	3 key strategic assets: employee know-how, organizational culture, and reputation. Reputation can be measured through customer satisfaction and number of high-profile awards achieved. Other IC info may be

			assets are capabilities and relationships within a firm, including employees, customer relationships, unique technologies, and intellectual property such as trademarks and brands. Strategic assets are intangible assets that are simultaneously valuable, rare, imperfectly imitable and non-substitutable.		GlaxoSmithKline Consumer Healthcare	gleaned from published communications about the organization and annual reports.
Sillanpaa et al., 2010*	The role of intellectual capital in non-profit elderly care organizations	To investigate the role of IC in the management of non-profit elderly care organizations	"an organization's non-physical sources of value" (Lev, 2001); consists of human, structural and relational capital	No explicit underlying theory; draws from literature on IC	Interviews with 6 managers in three organizations in the elderly care sector in Finland	Important IC resources: skilled personnel, working environment/atmosphere, reputation/brand, partners and networks, management (low hierarchy), organizational values. Current IC practices: customer satisfaction and needs assessments, internal audits, quality management, employee surveys, performance measurement. IC needs: more info on employee needs and competencies, info on reputation, info on long-term outcomes, process measures.
Wall, 2005*	The measurement and management of intellectual capital in the public sector	To determine whether public sector organizations are measuring and management IC	No explicit definition; IC used interchangeably with "intangible assets"; consists of human, organizational and customer capital. IC Management described as proactive and involving the identification and auditing of an inventory of IC assets as well as a continiuous evaluation of the value added by IC (Lynn, 1998)	No explicit underlying theory; draws from literature on IC and uses IC as a theoretical framework	Questionnaire with 57/100 leading public sector organizations in Northern Ireland in government, education and health (57% RR). 7/12 health institutions and boards participated.	86% were familiar with term IC, but only 14% have someone working on IC (none explicit). 11 have an ideas database; 8 maintain HR databases, two include records of skills, competencies, training, stakeholder etc. All IC elements viewed as important by at least 85%. Most important IC identified as reputation and customer satisfaction. Common HC measures: employee satisfaction, years of service and training. Common SC measures: customer satisfaction and complaints. Common OC measure: best practice (but still very low overall, 54%). OC least measured. None using traditional IC frameworks; most use "Investors in People"

						and "Balanced Scorecard". Lack of overall strategy for IC management.<br>Limitation: unable to extract results that pertain exclusively to the health institutions (cannot verify if they were health services organizations)
Weston et al., 2007	Reaping benefits from intellectual capital	To describe how nurse leaders can create the culture and infrastructure for reaping benefits of IC	No explicit definition provided, but implies that IC is synonymous with "knowledge resources"	No explicit underlying theory; draws from literature on IC and learning	Discussion paper	To create a culture to capture and embed IC requires: employee commitment, learning organization, social networks for sharing info, and employee participation in decision-making. Infrastructure support for IC includes social networks, IT, evidence-based practice approach, customization of best practices.
Wu & Hu, 2012*	Examining knowledge management enabled performance for hospital professionals: A dynamic capability view and the mediating role of process capability	To propose and empirically test a conceptual model linking hospital knowledge resources, hospital process capabilities, and hospital performance	"Knowledge assets [that are] inputs to an organization's value-creation process, which allow the organization to create and refresh its competencies over time"; consists of human, organization, and information capital	Resource-based view of the firm and knowledge-based view of the firm; draws from literature on knowledge management and dynamic capabilities	Questionnaire with 160 hospital executives or IT managers (33% RR) in Taiwan	Knowledge resources (assets & capabilities) played a role in knowledge management (knowledge management)-enabled hospital performance. Human and organizational capital more important to formation of knowledge assets than information capital. Found an interactive relationship between knowledge assets & capabilities; a mediating role for knowledge capabilities in enhancing process capabilities through knowledge assets; and a mediating role for process capabilities in improving performance through knowledge resources. Knowledge capabilities more significant than knowledge assets. Process capabilities predict both financial and patient performance. Outside-in capability more important to process capabilities than inside-out and spanning capabilities.
Yang & Lin, 2009*	Does intellectual capital mediate the relationship between HR management and organizational	To determine whether IC mediates the relationship HR practices and organizational	"intellectual material – knowledge, information, experience, core technique, intellectual property, and customer relationships – that	Resource-based theory; human capital theory	Questionnaire with 277 HR managers (56% RR) from 144 hospitals in Taiwan	Human, organizational and relational capital mediate the relationship between human resources management (HRM) practices and organizational performance (perceptual measures of employee satisfaction, patient

	performance? Perspective of a healthcare industry in Taiwan	performance; to explore what HR practices can best explain IC	can be put to use to create wealth" (Stewart, 1997); three components: human, organizational and relational capital. HC: "all knowledge, skills and experience of both employee and manager." OC: "institutionalized knowledge and codified experience stored in databases, routines, patents, manuals, structures, and the like". RC: "knowledge resources embedded within, available through, and derived from networks of relationships between peers, customers, suppliers, and business associates" (Bontis 1998; Stewart 1997, Youndt & Snell 2004)			loyalty, turnover rate, and quality care). Five HRM practices contribute to accumulation of IC, and IC explains organizational performance (especially organizational capital which had two times the predicting power). Recruitment and selection, and health and safety, linked to all 3 types of IC. Performance appraisal linked to organizational and relational capital. Training and development linked to human capital. Compensation had no effect on IC in explaining organizational performance.
Zigan et al., 2008*	Intangible resources as performance drivers in European hospitals	To explore managers' perceptions of importance and impact of intangible resources, and the role/use of intangible resources in performance management of Euro hospitals	"holistic or meta-level capability of an enterprise to coordinate, orchestrate and deploy its knowledge resources to create value in pursuit of its future vision" (Rastogi, 2003); consists of human, structural or organizational, and relational or customer capital; use IC and "intangible resource" interchangeably	No explicit underlying theory; draw from literature on IC and performance measurement in healthcare; use IC as a conceptual framework for the study	Semi-structured interviews with 6 representative from hospitals in Norway, Germany and UK	Respondents agreed on the importance of intangible resources. Only few hospitals had a formal system in place for development of these resources. Focus seemed to be on human capital with some recognition of structural capital (IT) and relational. HR systems identified as weak. Relational capital viewed as relationships with patients, different professional groups, and other hospitals. Lack of individual performance measurement. Sharing IC viewed as positive.
Zigan et al. 2009*	The Identification of Important Intangible Resources in Hospitals	To identify what managers perceive to be key intangible resources used in hospitals, and to	Uses the terms intangible resources and IC interchangeably. No explicit definition provided, just examples such as knowledge,	Resource-based view of the firm; also draw from literature on knowledge generation and sharing	Semi-structured interviews with admin managers(n=14) and clinical staff (n=8) in a German university hospital	Examples of intangible resources provided that span three categories: human, structural, social and relational. Human capital identified as the most important, alongside social capital and staff attitudes

		explore whether known intangibles are managed in a systematic way	relationships, communication			towards the organization and towards change. Hospital characteristics linked to types of IC: knowledge-intensive (human capital), complex and bureaucratic (structural capital), various occupational groups (social capital), and numerous stakeholders (relational capital)

* Empirical paper
